# Supplementary material for: Novel and improved Caenorhabditis briggsae gene models generated by community curation
Source: bioRxiv. 2023 May 18:2023.05.16.541014. Preprint. [Version 1] doi: 10.1101/2023.05.16.541014 (PMC10245686; doi:10.1101/2023.05.16.541014)
Supplement: Supplement 2 [file media-2.pdf]

Caenorhabditis briggsae gene annotations improved by community curation using short- and long-read RNA sequencing

Nicolas D. Moya[1,2], Isabella R. Miller[1], Chloe E. Sokol[1], Joseph L. Galindo[1], Alexandra D. Bardas[1], Edward S. H. Koh[1], Justine Rozenich[1], Cassia Yeo[1], Maryanne Xu[1], and Erik C. Andersen[1,‡]

1. Department of Molecular Biosciences, Northwestern University, Evanston, IL 60208, USA
2. Interdisciplinary Biological Sciences Program, Northwestern University, Evanston, IL 60208, USA

Supplemental Information 2

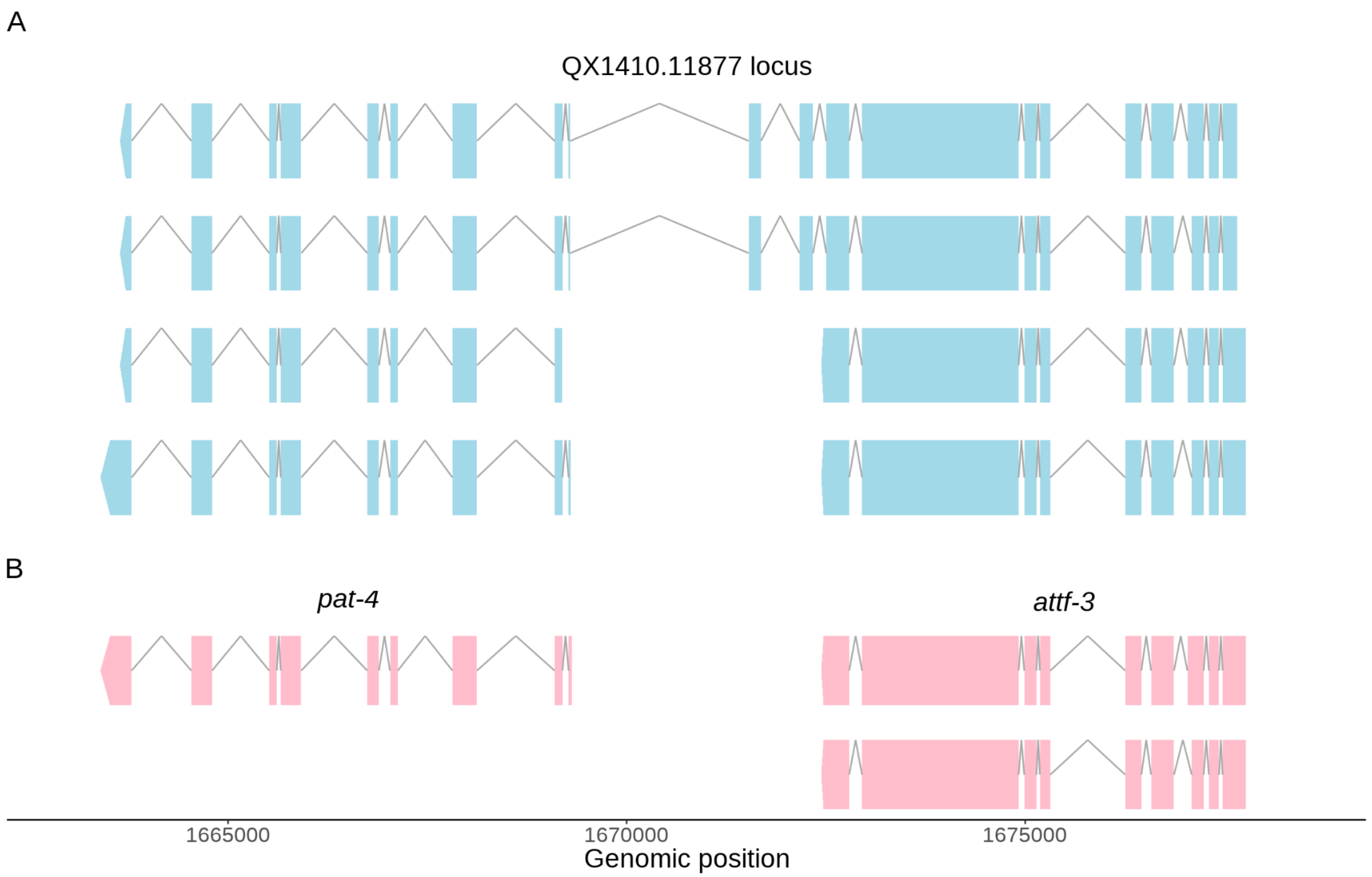

Supplemental Figure 1: Example of BUSCO duplication reduction after manual curation
Plot showing the gene models located on chromosome III from 1,663,398 to 1,677,797 before (A) and after (B) curation. Six software-derived models are shown in blue and are annotated as isoforms of a single gene. Three spurious isoforms were removed during the curation process, and the locus was divided into two separate genes, orthologous to the *C. elegans pat-4* and *attf-3* genes. The *pat-4* gene is part of the BUSCO database, and it is no longer duplicated. The *attf-3* gene is composed of two isoforms that have differences in the 3' termini of exon 3.

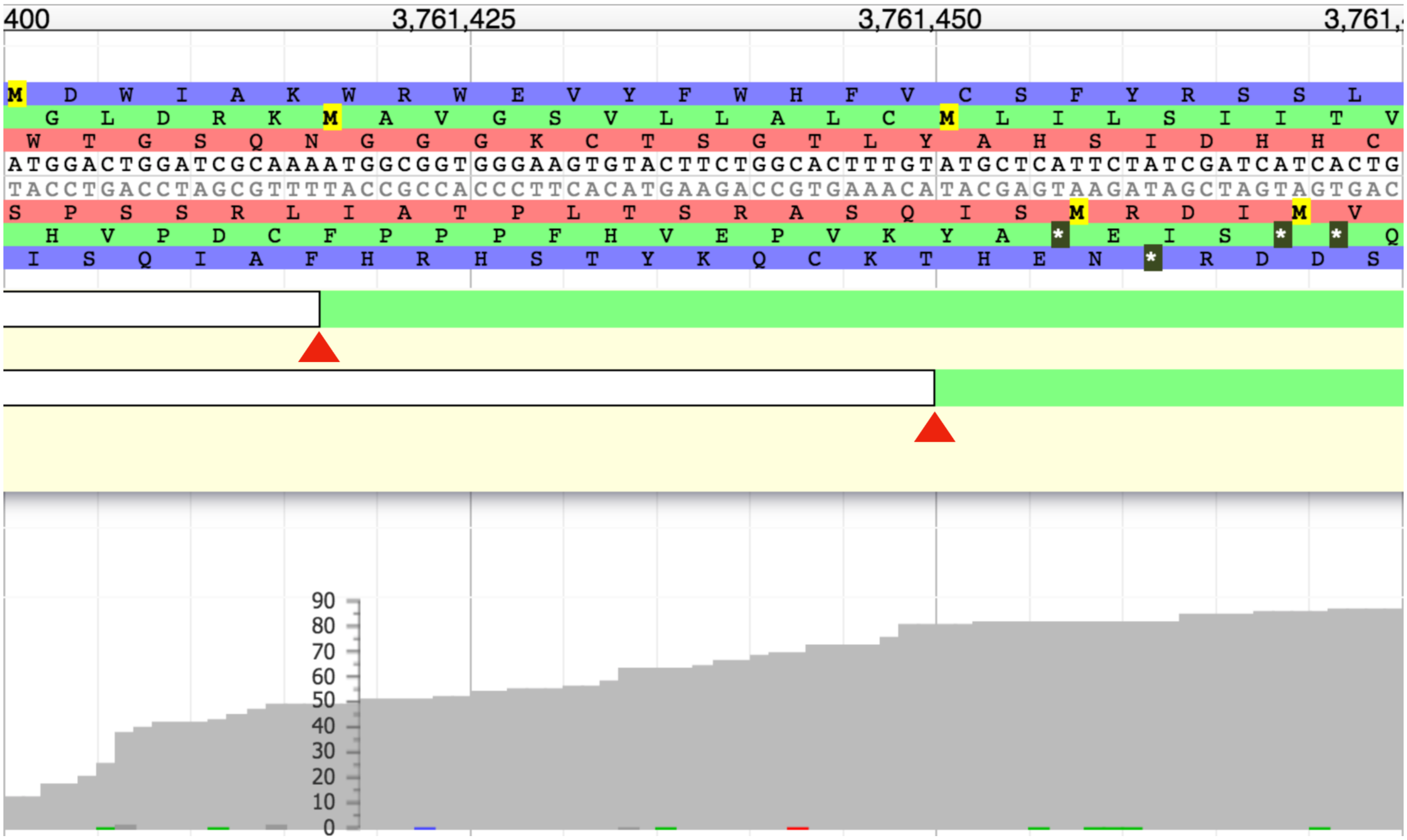

Figure S2: Example of alternative translation initiation site

Screen capture showing a genomic window of 75bp (located on chromosome III from 3,761,400 to 3,761,475) that covers the 5' termini of the *gar-2 C. briggsae* ortholog. Two alternative in-frame translation initiation sites are highlighted with red arrows. Both sites have similar RNA coverage (shown in gray).

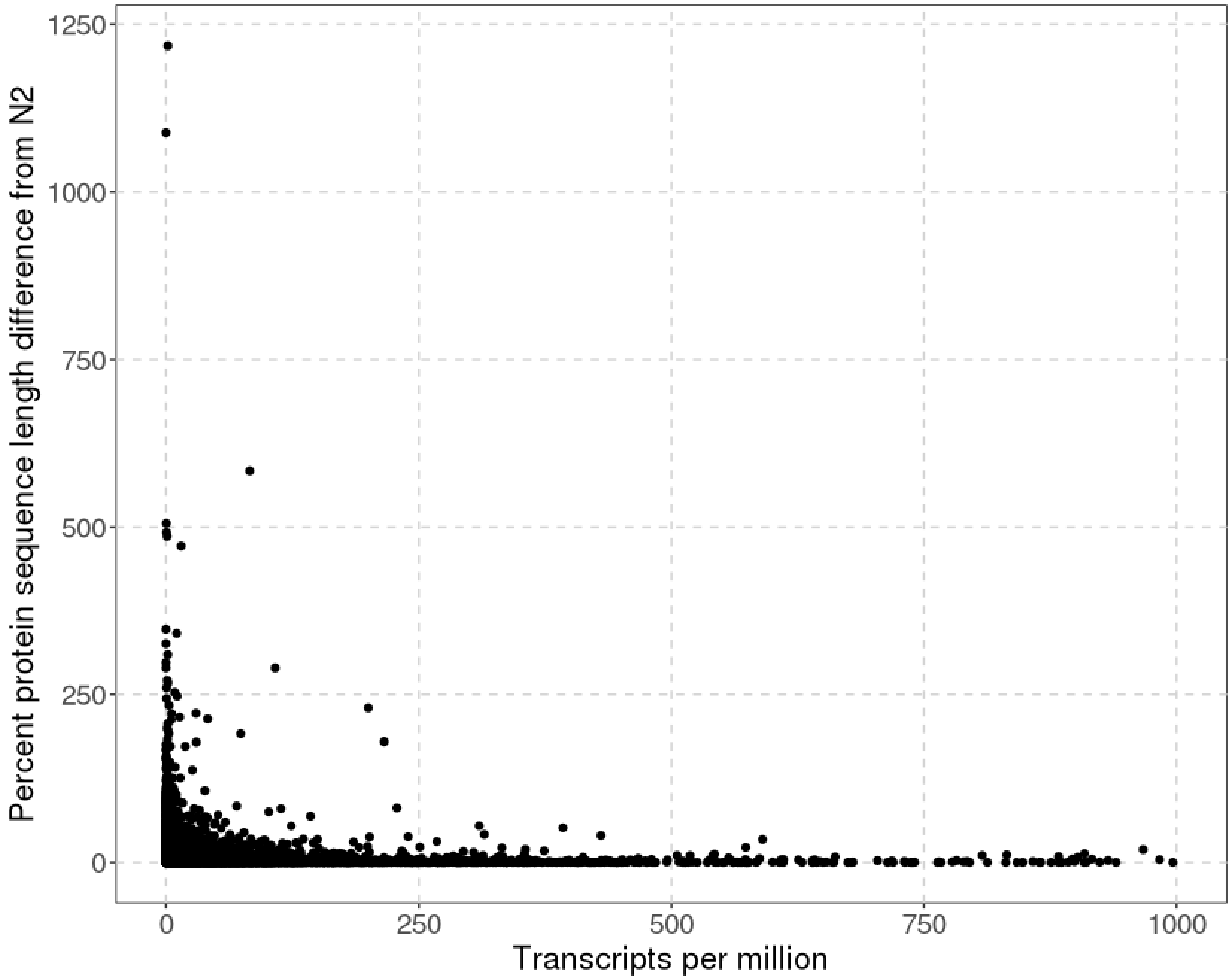

Figure S3: Protein-length accuracy of QX1410 genes by TPM
Plot showing the transcripts per million for each QX1410 gene against its percent protein length distance to its N2 ortholog.

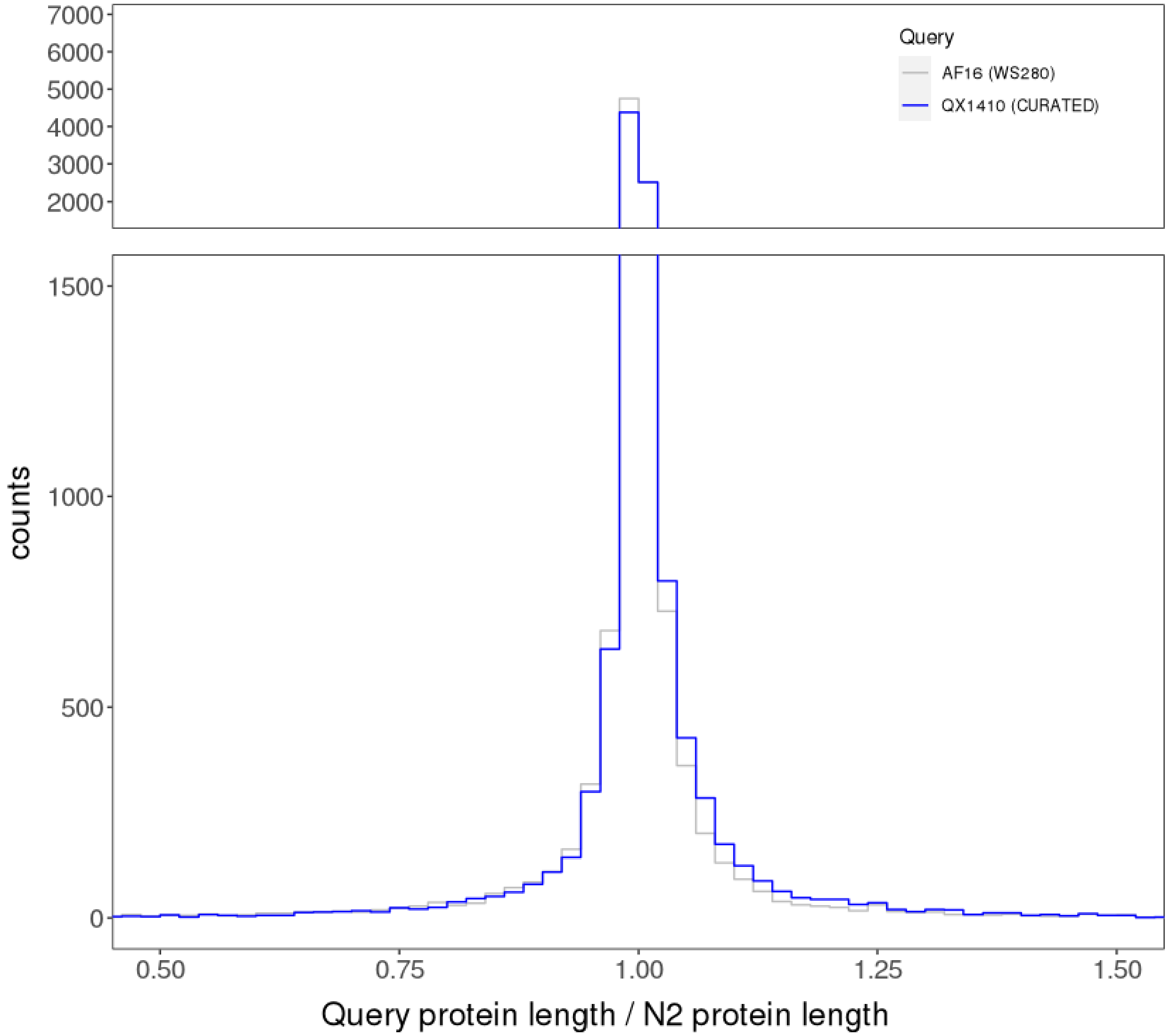

Figure S4: Protein length accuracy of *C. briggsae* gene models relative *C. elegans* N2 inferred single-copy orthologs

We calculated the protein length accuracy of both curated QX1410 gene models and AF16 (WS280) gene models using single-copy orthologs identified through phylogenetic orthology analysis. A ratio of one represents a *C. briggsae* gene with a protein length that perfectly matches its single-copy ortholog in N2.

Table S1: Protein-length accuracy and BUSCO completeness before and after revision of 755 loci with deviations in protein length

	Before revision	After revision
one-to-one matches to N2	2601	2710
5% off matches to N2	7849	7940
BUSCO completeness	99.7%	99.7%

Table S2: Protein-length accuracy using phylogeny-inferred orthologs between QX1410, AF16, and N2.

	AF16	QX1410
one-to-one matches to N2	2222	2173
5% off matches to N2	8762	8811

Table S3: Intra-chromosomal duplications in the AF16 genome and affected genes

segment A	segment B	identity	genes in segment A	genes in segment B
I:12895-14211	I:396029-397345	99.57	Cbr-rps-29	CBG11412
I:175947-178569	I:169983-172605	99.7	CBG11861, CBG00361, CBG11492	Cbr-arrd-2, Cbr-sbds-1, CBG11493
I:134401-138262	I:126814-130653	99.77	CBG01029, CBG15263, Cbr-ncs-3	Cbr-lim-6
I:124226-127163	I:131671-134750	99.42	CBG30931, Cbr-pfn-3	CBG15264
I:50373-56695	I:42341-48591	99.62	CBG01049, CBG01048, CBG01046, CBG11525, CBG11524	Cbr-glt-5, CBG00337, Cbr-fmo-4
I:1171622-1178466	I:1218277-1225147	96.33	CBG16333	CBG18556, CBG00846, CBG00845, Cbr-hpo-38, Cbr-acr-15, CBG16326
I:1352610-1357683	I:1361512-1366585	99.22	CBG00754, CBG27416	CBG15737, CBG27418
I:1463157-1465588	I:1466607-1469038	97.69	CBG00785	CBG31329, CBG00786
I:1678479-1681854	I:1700745-1704120	99.27	CBG25180	CBG30327
I:1807933-1810419	I:1811400-1813886	96.44	CBG30809	CBG22020, CBG08816
I:2001351-2004701	I:2006517-2009867	97.19	CBG21555, CBG21557, CBG31291	CBG21559
I:2006686-2009893	I:2001512-2004758	97.81	CBG21559	CBG21557, CBG31291
I:3219715-3226441	I:2424060-2430786	99.9	CBG29818, CBG11638, CBG17031, CBG30501, Cbr-tmem-218	Cbr-pqn-59.1, CBG30439, CBG26638
I:2797516-2798692	I:2802327-2803504	99.83	CBG22202	CBG22203
I:3208166-3215423	I:3275534-3282791	99.72	Cbr-smo-1, CBG22302, Cbr-glna-2, CBG26833	CBG15102, CBG15103, CBG15104, CBG13342, CBG10509, CBG10508
I:3456270-3466382	I:3456270-3466382	99.98	Cbr-xrep-4, Cbr-trpp-8, CBG14903, CBG00725, CBG24735, Cbr-pes-1, CBG10454, Cbr-gnrr-3	Cbr-xrep-4, Cbr-trpp-8, CBG14903, CBG00725, CBG24735, Cbr-pes-1, CBG10454, Cbr-gnrr-3
I:3560841-3566940	I:3567941-3574040	99.8	Cbr-elo-8, Cbr-srg-3, CBG17473	CBG30964, CBG22594, CBG10425, CBG01955
I:4963727-4964839	I:15222377-15223491	94.92	CBG03918	CBG17036
I:5594552-5601685	I:5606871-5614004	99.98	CBG03762, CBG03761, CBG03760, Cbr-fip-3.1, CBG18231, Cbr-exos-1, CBG17865, CBG30898	CBG03699, Cbr-cel-1, CBG17869, CBG17870
I:8006840-8008057	I:12076549-12077849	85.01	CBG31390	CBG00002
I:5779531-5781829	I:5830921-5833219	99.91	CBG30597, Cbr-ttr-31	CBG27553

I:1673321-1674689	I:13084727-13086094	99.78	CBG26161	CBG16717
I:6706546-6707749	I:6708365-6709568	99.83	Cbr-mzt-1.2	Cbr-mzt-1.1, CBG03284
I:6867179-6869525	I:6858657-6860983	92.55	Cbr-wee-1.1, CBG06567	CBG21906, Cbr-cnc-4, Cbr-cnc-3
I:6858655-6860982	I:6867180-6869526	92.64	CBG21906, Cbr-cnc-4, Cbr-cnc-3	Cbr-wee-1.1, CBG06567
I:7884100-7891151	I:7028193-7035244	99.97	CBG12104, CBG12105, Cbr-viro-2, Cbr-pot-1, CBG26353, CBG05826, Cbr-swt-7	CBG11902, CBG11903, CBG30143, Cbr-paqr-1, CBG06610, Cbr-nhr-178, CBG06612
I:7233822-7241056	I:7245702-7252936	99.96	Cbr-spe-4, Cbr-fut-1, CBG17973, CBG26323, Cbr-adk-1, CBG27367, CBG27601	CBG11945, CBG11946, Cbr-tars-1, CBG06026, Cbr-rap-3
I:7276238-7279267	I:7892152-7895182	100	CBG03133, CBG17962, CBG17136	CBG26592, CBG27617
I:7338216-7343793	I:7344751-7350328	99.6	CBG25559, CBG30033, CBG05994, CBG05993, CBG17119	Cbr-flap-1, CBG06687
I:7523170-7526884	I:7527845-7531559	99.95	CBG12018, Cbr-wip-1, CBG11828	CBG06740
I:8486251-8487568	I:8484615-8485932	99.85	CBG29853	CBG29852
I:8484614-8485932	I:8486251-8487569	99.92	CBG29852	CBG29853
I:8433926-8435390	I:8005537-8007001	99.84	CBG14192	CBG02960
I:8002165-8007001	I:8427483-8432320	99.44	CBG23931, Cbr-pdp-1.1, Cbr-rps-9, CBG02961, CBG02960, CBG05790, CBG08616	CBG12192, Cbr-pdp-1.2, CBG23522
I:8369874-8372703	I:8364987-8367816	99.92	CBG12206, CBG06038	CBG12207
I:8146914-8148994	I:8712277-8714346	87.66	CBG25949	CBG12411
I:8048439-8057284	I:8027971-8036816	99.95	CBG02255, CBG02254, CBG02253, Cbr-bigr-1, Cbr-lir-3, CBG02950, Cbr-str-48, CBG08598, CBG08597	CBG24754, Cbr-hsp-12.1, CBG24756, CBG24757, Cbr-pqm-1, CBG08607, CBG30625, CBG08605
I:8712279-8714362	I:8146901-8148995	87.7	CBG12411	CBG25949
I:9828246-9833872	I:9835223-9840863	99.98	Cbr-nuo-2, CBG30979, Cbr-dhp-2, CBG03445	CBG29118, CBG12673, CBG26395
I:10596874-10605615	I:10606616-10615357	99.96	Cbr-txt-9.1, CBG02319, Cbr-ptr-9, CBG01749, CBG01748, Cbr-set-22, Cbr-snf-10, Cbr-pap-1	CBG02316, CBG31350, CBG02315, Cbr-nhl-1, CBG01746, Cbr-rpl-20, Cbr-cyc-2.1, Cbr-tctn-1, CBG09606
I:11017779-11018528	I:11026776-11027525	99.8	Cbr-msrp-1	Cbr-msrp-6
I:11035842-11039634	I:11078044-11081817	97.83	CBG20251, CBG01632	CBG20242, CBG30532, CBG09452

I:11078047-11081816	I:11035857-11039634	97.81	CBG20242, CBG30532, CBG09452	CBG20251, CBG01632
I:11080655-11081727	I:11231511-11232585	89.94	CBG20242	CBG08500
I:11399713-11403810	I:11571154-11575259	99.12	Cbr-hpo-15	CBG13610, CBG17707, Cbr-str-27, Cbr-odd-2
I:11571173-11575235	I:11399712-11403810	98.95	CBG13610, CBG17707, Cbr-odd-2	Cbr-hpo-15
I:12365185-12369064	I:12507990-12511869	99.99	CBG20401, CBG20402, CBG10926	CBG20432, CBG20433, CBG19627
I:13001070-13005877	I:3457-8335	95.38	CBG19165	Cbr-sqv-8, CBG01066, CBG00325, Cbr-grl-17
I:12722206-12726069	I:12837614-12841445	99.17	Cbr-fipr-24.2, CBG30066, Cbr-map-1	CBG18839
I:12548002-12554217	I:13061341-13067525	99.24	CBG19620, CBG08368, Cbr-odc-1, CBG30805	CBG30069, CBG19495, CBG21194, CBG19186, CBG19187, CBG19188, CBG27815
I:13812723-13815134	I:13809420-13811856	99.59	CBG18489	CBG30593, CBG19371, CBG16882
I:13809433-13811847	I:13812715-13815148	99.88	CBG19371, CBG16882	CBG18489
I:14796277-14799080	I:14801167-14803970	95.19	CBG04652	CBG04655
I:15222383-15223530	I:4963727-4964841	94.57	CBG17036	CBG03918
I:15257988-15265658	I:15249949-15257621	99.79	CBG04781	CBG16021, Cbr-tbx-34, CBG31278, Cbr-mtl-2, CBG04780
II:66677-71747	II:72748-77818	99.86	Cbr-sde-2, CBG11515, CBG08049	CBG00342
II:512064-515307	II:515675-518922	93.72	CBG13813, Cbr-egl-17	CBG26871
II:1216389-1220711	II:1133917-1138239	99.06	CBG30967, CBG00846, CBG15718, CBG16326	CBG00857
II:1121882-1129102	II:1199474-1206680	98.45	CBG18542, Cbr-sfxn-1.2, CBG30324, Cbr-oac-29, CBG16345	CBG30969, Cbr-taco-1, CBG15715
II:1488444-1491181	II:1484260-1486997	99.62	CBG00797	CBG00795
II:1595824-1596950	II:1611602-1612728	99.3	CBG26157	CBG26158
II:1592359-1596028	II:1612521-1616190	99.84	CBG16241	CBG05101, CBG00839, CBG00840, CBG27433
II:1778662-1781943	II:1855128-1858523	99.76	CBG18444, CBG29999, Cbr-josd-1, CBG00487	CBG31002
II:1989928-1991960	II:1994663-1996695	95.07	CBG29812	CBG08875
II:2314636-2315656	II:13733152-13734152	94.26	CBG31124	CBG19348
II:2426866-2430072	II:2434507-2437713	97.35	CBG30439, CBG26638	CBG30496
II:2865336-2870299	II:2901580-2906538	100	CBG25484	CBG22230, CBG13419, CBG13418, CBG22362,

				Cbr-srj-7, Cbr-srj-11, CBG15947
II:2880915-2884271	II:3069972-3073328	99.94	CBG13424, CBG22357, Cbr-srj-9	CBG22271, CBG13380, CBG13379, CBG16983
II:2885288-2892119	II:3262407-3269238	99.63	CBG13423, CBG25829, CBG22359, CBG22421, Cbr-sdz-34.4	CBG15100, CBG13343, Cbr-nmt-1, CBG10514, CBG01894
II:4158933-4168350	II:4314672-4324083	99.64	CBG04167, CBG00542, CBG00543, CBG09040, CBG09039, Cbr-lipl-5, CBG26990, CBG26989, Cbr-nlp-34.4, CBG26988	Cbr-cep-1, Cbr-rpl-25.2, CBG04079, Cbr-wrm-1, CBG30196, CBG26982, CBG26981, CBG02102, CBG02103
II:4144222-4148435	II:4139998-4144211	98.55	CBG00540	CBG04172, Cbr-dnc-6, CBG31324, CBG00538, Cbr-erd-2.2
II:4823536-4828914	II:4834010-4839388	99.63	Cbr-coa-4, CBG01159	CBG04252, CBG31007, CBG31179, CBG21694, Cbr-srj-33, CBG30816
II:4838194-4839388	II:4839399-4840597	98.25	CBG21694	CBG04254
II:4842262-4845929	II:4830114-4833782	99.89	CBG01152, Cbr-ftt-2	CBG01158
II:5304613-5308357	II:5308458-5312191	99.97	CBG12122, CBG12123	CBG11131, Cbr-hex-3, CBG25998, CBG12124
II:5819690-5824660	II:5825661-5830631	99.98	Cbr-stl-1, CBG13080, CBG13183, CBG17917, CBG27385	CBG17919, CBG17920, Cbr-col-177
II:6564597-6570699	II:6576189-6582283	99.98	CBG10822, CBG10823, CBG10824, Cbr-cki-2, CBG18137, CBG30316, CBG27086, Cbr-str-46, CBG06463	Cbr-mat-1, CBG12945, Cbr-pqn-96, Cbr-ufbp-1, Cbr-ifc-1, Cbr-str-39
II:6572050-6576178	II:6582297-6586425	99.99	CBG12946, Cbr-srw-4, Cbr-srw-3	CBG10829, Cbr-pfkb-1.1, Cbr-rpn-3, CBG04479
II:7392256-7393219	II:9662010-9663024	91.8	CBG30348	CBG30738
II:7470239-7475817	II:7484413-7489991	99.64	CBG25565, Cbr-clec-61, CBG05950, CBG05949, Cbr-cpz-2, CBG11812, CBG26804	Cbr-unc-108, CBG03093, CBG03092, CBG25922, Cbr-nhr-8, CBG30726
II:9662013-9663056	II:7392255-7393260	91.6	CBG30738	CBG30348
II:10052028-10056936	II:10057956-10062864	99.96	Cbr-mdt-26, Cbr-ift-43.1, Cbr-rpl-25.1	CBG12716, CBG02468, Cbr-ift-43.2, Cbr-scl-1, Cbr-scl-9
II:10109241-10116736	II:10119713-10127208	99.95	CBG29868, CBG12731, Cbr-cdc-14.2, Cbr-jmjd-3.2, Cbr-hum-10	CBG12733, CBG12734, Cbr-ttc-4, Cbr-cdc-14.1, CBG25640, CBG24368, CBG24369, CBG24371, CBG09743, CBG30526, CBG27068

II:10185727-10189076	II:10192435-10195784	99.79	CBG02428, CBG26018, CBG06900	CBG12747, CBG02426, CBG14554
II:10189076-10191758	II:10198301-10200983	99.95	CBG02427, CBG06899	CBG25369, CBG02425, CBG06896
II:10198299-10200981	II:10189076-10191760	100	CBG25369, CBG02425, CBG06896	CBG02427, CBG06899
II:10281451-10283198	II:10161077-10162798	96.56	CBG02400	Cbr-sec-61.G
II:11375377-11381801	II:11385306-11391730	99.72	CBG08467, CBG08466, CBG25403, CBG20787, CBG20788, CBG14803	CBG08462, CBG25402, CBG20790, CBG20791, CBG09383
II:12296859-12303234	II:12303245-12309620	98.92	Cbr-mtx-2, CBG00034, CBG30446, CBG18975, CBG18976, Cbr-asg-2	Cbr-csn-4, Cbr-gon-14, CBG10907, CBG10908
II:11667151-11673571	II:11680357-11686777	97.8	CBG20860, Cbr-mvb-12, CBG09304, CBG05010	CBG26030, CBG20145, Cbr-ric-3, CBG09301, CBG05015
II:11930967-11935132	II:12407061-12411226	99.8	CBG30118	CBG20410, CBG20411, CBG31464, CBG30650
II:12416274-12422208	II:11935143-11941077	99.86	CBG20414, CBG12246, Cbr-grl-8	CBG17611, Cbr-sdz-13, CBG05074, CBG05075
II:12076340-12077955	II:11933037-11934598	97.26	CBG00002	CBG30118
II:12264279-12266701	II:12423299-12425723	93.86	CBG20995	CBG31280
II:12427940-12429153	II:12430973-12432180	87.31	CBG21037	CBG21039
II:12430977-12432176	II:12427936-12429154	88.91	CBG21039	CBG21037
II:12908766-12912742	II:12439803-12443779	99.94	CBG31528	Cbr-ufm-1
II:12444663-12450908	II:12914549-12920794	99.84	Cbr-rpl-35, Cbr-ugt-59, CBG19012, Cbr-bbs-8	CBG29933, CBG31529, CBG19532, CBG30250, CBG21224, CBG26479, CBG26478, CBG11046
II:12453480-12457714	II:12459869-12464047	98.02	CBG20418, CBG19643, Cbr-srh-30, Cbr-srh-28	CBG19641, Cbr-ugt-44, Cbr-spn-4, Cbr-tsp-16
II:12459833-12464050	II:12453480-12457672	96.3	CBG19641, Cbr-ugt-44, Cbr-spn-4, Cbr-tsp-16	CBG20418, CBG19643, Cbr-srh-30, Cbr-srh-28
II:12466977-12473539	II:12931949-12938511	99.71	CBG20421, CBG19638, CBG08387, CBG19020	CBG21221, Cbr-str-84, CBG19146, CBG11049
II:13018194-13023072	II:13011860-13016763	99.84	CBG19507, CBG19171, CBG30543, CBG19173, Cbr-swm-1	CBG18879, CBG19510, CBG26473, Cbr-nhr-30.1, CBG27177
II:13101976-13105215	II:13106002-13109244	96.42	CBG19486, Cbr-sri-40, CBG10726, CBG19199, CBG19200	CBG19483, CBG26165, CBG21186
II:13106002-13109823	II:13101394-13105215	99.61	CBG19483, CBG26165, CBG21186, CBG30547, Cbr-col-174	CBG19486, Cbr-sri-40, CBG10726, CBG30546, CBG19199, CBG19200
II:13184527-13187950	II:13188328-13191751	99.95	CBG21174, CBG31168	Cbr-atp-2

II:13195818-13199676	II:13165668-13169526	99.94	CBG04349	CBG29899
II:13210515-13212263	II:13201048-13202824	97.81	Cbr-nlp-61	CBG04350
II:12099401-12100628	II:14523348-14524575	92.86	CBG20948	CBG21869
II:14523348-14525105	II:14527276-14529035	93.09	CBG21869	CBG24810
II:14526707-14528432	II:15101545-15103253	89.57	CBG24810	CBG17057
II:15101738-15103253	II:14203501-14205016	87.68	CBG17057	CBG10347
II:14203502-14204860	II:14420582-14421965	92.36	CBG10347	CBG10283
II:14420582-14421793	II:12099489-12100654	86.52	CBG10283	CBG20948
II:13733152-13734152	II:2314652-2315656	95.24	CBG19348	CBG31124
II:13973009-13974032	II:13984297-13985291	86.54	Cbr-ins-32.2	Cbr-prx-11, Cbr-ins-32.5
II:14150197-14151510	II:14143404-14144716	98.17	CBG07912	CBG07911
II:14557696-14560000	II:14568356-14570614	94.38	CBG24149	CBG16447, CBG31150, CBG04587
II:14808172-14809274	II:14871003-14872119	97.77	CBG21442	CBG21457
II:15294684-15296640	II:15284353-15286275	95.88	CBG16034	Cbr-flp-33, CBG16029
II:15284353-15286278	II:15294670-15296630	97.96	Cbr-flp-33, CBG16029	CBG16034
II:16600308-16607497	II:16607799-16614988	99.97	CBG06958, Cbr-drl-1, Cbr-srxa-7	Cbr-dhs-6, CBG30393
III:251831-254547	III:259312-262037	99.93	CBG15234	CBG11878, CBG00994
III:332180-336194	III:336342-340356	99.93	CBG11438, CBG11437	Cbr-srx-42
III:528787-534740	III:534751-540704	98.74	Cbr-str-144	Cbr-catp-3
III:958113-962780	III:1627831-1632509	99.96	CBG11267	CBG15793, CBG08765, CBG16229
III:1649736-1656389	III:1144021-1150674	99.92	Cbr-gsto-3.1, CBG15798, Cbr-srt-44.1, CBG13846, CBG16223	Cbr-gsto-3.2
III:1658437-1664270	III:1538017-1543850	99.94	CBG05095, CBG31062, CBG13848, Cbr-mfsd-11	Cbr-psf-3, CBG29807, CBG00814
III:6811048-6814310	III:1755904-1759159	99.72	CBG21890, CBG30952	CBG00479
III:1880950-1883530	III:1885400-1888002	97.36	CBG22029	Cbr-spp-23
III:3406569-3412108	III:3132111-3137650	98.45	CBG11677, CBG27477	CBG17009, Cbr-sup-10
III:3177475-3184954	III:3184965-3192444	99.52	CBG22296, CBG21090, CBG31277, CBG21093, Cbr-str-164, CBG30649, CBG17019	CBG21094, CBG31276, Cbr-str-163, CBG31037, CBG17021, Cbr-lec-9
III:3936476-3944683	III:3822463-3830670	99.96	CBG04123, CBG04124, CBG29824, Cbr-tofu-4, CBG09097, CBG01426, CBG01425	Cbr-itx-1, CBG09118, Cbr-cutl-23, Cbr-cav-1
III:3940131-3944683	III:3945684-3950236	99.74	CBG29824, Cbr-tofu-4, CBG01425	Cbr-mage-1, CBG09096
III:3983939-3985307	III:4118576-4119916	90.88	CBG09091	CBG09051, CBG30336

III:4529767-4531348	III:4496830-4498393	84.98	CBG05212	CBG08958
III:4557040-4562953	III:4517123-4523036	99.91	Cbr-kbp-3, CBG26141, CBG01239	Cbr-ceh-12, Cbr-col-54, Cbr-nlp-62, CBG30197, CBG21752, CBG21751, CBG01253, Cbr-sav-1
III:4629492-4631486	III:4624163-4626157	99.94	CBG05182	CBG01217
III:4792753-4797708	III:4797809-4802764	99.93	CBG04244, CBG23800, CBG29116, CBG01169	Cbr-ept-1, CBG25513, CBG24620, CBG21708, CBG01168
III:5008803-5010747	III:5130826-5132779	99.28	CBG18376	CBG18345
III:5130808-5132791	III:5008794-5010746	99.23	CBG18345	CBG18376
III:5195994-5198512	III:4989036-4991546	100	CBG18325	Cbr-mrpl-12, CBG18379, CBG01113
III:5570162-5575549	III:6044340-6049727	99.29	Cbr-ikb-1, CBG18240	Cbr-edg-1, CBG13233, Cbr-srbc-58
III:5573187-5575346	III:6049734-6051898	97.56	CBG18240	CBG13234
III:6049737-6051898	III:5573185-5575346	99.78	CBG13234	CBG18240
III:5793019-5797080	III:5798081-5802142	99.97	Cbr-npr-31, CBG30598	CBG30025, Cbr-fust-1
III:5883416-5890760	III:5891471-5898815	99.97	Cbr-bro-1, CBG26571, CBG13099, CBG26150, CBG30027, CBG13100, CBG30843, CBG19948, Cbr-srx-22, Cbr-irld-3, CBG17543, Cbr-nlp-19	Cbr-npp-6, CBG13103, CBG26414, CBG06281, CBG06282, CBG17546
III:6351606-6359943	III:6364385-6372722	99.97	Cbr-pgam-5, CBG13314, Cbr-trap-4.1, CBG13317, CBG06415, CBG06416	Cbr-paf-1, CBG29840, CBG13319, Cbr-ccf-1, Cbr-trap-4.2, CBG13322, Cbr-ges-1, CBG06419
III:6503869-6505020	III:6505647-6506829	95.88	CBG18155, Cbr-rps-25	CBG18154
III:6505649-6506802	III:6503867-6505047	95.88	CBG18154	CBG18155, Cbr-rps-25
III:6809503-6814263	III:1754325-1759159	98.58	CBG18081, CBG30952, Cbr-srv-24	CBG30277, CBG30278, CBG00479
III:7871256-7875249	III:7875575-7879568	99.97	CBG31459, CBG08655	CBG16596, CBG08654, CBG08653, CBG08652
III:9037020-9037720	III:9034754-9035454	99.88	CBG09918	CBG09917
III:9202714-9206259	III:9207273-9210818	99.67	CBG02683, CBG09964, CBG14340	CBG14341
III:9245530-9251347	III:9306132-9311949	99.91	Cbr-slc-25A42, CBG12502, Cbr-lmd-1, Cbr-rsp-5, Cbr-snr-3, Cbr-vps-53, CBG09979, CBG09980, CBG30353, Cbr-ppm-1.H, Cbr-rpl-34	CBG02655, CBG10002, CBG10004, CBG14364, CBG14365
III:9551641-9553240	III:9554139-9555738	99.64	CBG14427	CBG23216

III:9757716-9763607	III:9764252-9770143	99.96	CBG12647, CBG02546, CBG10121	CBG02545, CBG02544, CBG25631, CBG30848, CBG10123, CBG03424
III:10642341-10648145	III:10635536-10641340	99.96	CBG30842, CBG06793, Cbr-aqp-5	CBG12848, CBG02308, CBG06795, Cbr-bus-4, CBG09600, Cbr-vps-41
III:10613619-10620004	III:10606233-10612618	99.84	Cbr-ptp-5.3, CBG02313, Cbr-timm-17B.1, Cbr-hoe-1, Cbr-chw-1, CBG14647, CBG14648	CBG02316, Cbr-nhl-1, CBG01746, Cbr-rpl-20, Cbr-cyc-2.1, CBG30616, CBG09606
III:10573860-10576681	III:9960308-9963129	99.94	CBG02329, CBG27075	CBG29867, CBG23095
III:10157359-10162673	III:10163074-10168388	99.98	CBG25368, CBG06907, CBG24380, CBG09731, CBG27070, Cbr-sec-61.G	CBG02435, CBG06906, CBG24382, CBG24383, CBG09729
III:10234616-10235954	III:10236549-10237922	91.55	Cbr-sdz-1.2	Cbr-sdz-1.1
III:10236550-10237922	III:10234616-10235955	91.55	Cbr-sdz-1.1	Cbr-sdz-1.2
III:10480496-10483611	III:9956160-9959307	97.56	CBG27727, Cbr-dpy-8	Cbr-col-123, CBG23097
III:9950187-9954039	III:10462958-10466810	99.92	CBG23099	CBG12811, Cbr-pho-1, CBG14615
III:9956159-9959307	III:10480499-10483647	99.22	Cbr-col-123, CBG23097	CBG27727, Cbr-dpy-8
III:10864685-10868502	III:10868977-10872770	99.89	CBG20614	CBG20616, CBG03559, CBG27741
III:11685663-11689846	III:11689947-11694129	99.67	CBG20144, CBG17682, CBG05016	CBG31295, CBG09120, CBG27755
III:11758538-11763590	III:11765319-11770361	99.48	CBG13645, CBG30986	CBG17655, CBG17654, Cbr-cest-16
III:11908810-11913422	III:11916636-11921248	99.96	Cbr-rbm-3.1, Cbr-sdz-15, Cbr-nas-33	CBG18681, CBG18680, Cbr-nos-1
III:12529827-12540959	III:12542096-12553228	99.41	CBG19622, Cbr-tyr-1, CBG27775	CBG19621, CBG19620, CBG12272, CBG08369, CBG30957, CBG08368, CBG19039, Cbr-odc-1, CBG27777, CBG30805
III:12676844-12686619	III:12687621-12697405	99.94	CBG19589, Cbr-fut-4, CBG30065, Cbr-gsto-1.1, Cbr-egl-21, Cbr-bgnt-1.1, CBG19078, Cbr-nhr-286, Cbr-nhr-135	CBG18810, CBG26173, CBG19583, Cbr-gsto-1.2, Cbr-otpl-1, CBG11000
III:13666809-13676171	III:13685043-13694405	99.72	CBG15379, CBG26207, Cbr-srx-97, CBG23012, Cbr-cyp-29A4, CBG19333	CBG15382, CBG21425, Cbr-pitr-4, Cbr-sulp-8
III:14396033-14403741	III:14404742-14412450	99.8	Cbr-ztf-30.2, CBG04555, CBG04556, Cbr-srh-17	Cbr-impt-1, CBG08010, CBG10287, Cbr-ztf-30.1, Cbr-gasr-8

III:14513523-14521015	III:14246586-14254078	99.73	CBG08031, CBG26568, Cbr-acbp-4.2, CBG15352, CBG15353	CBG10336, CBG00606, CBG00607, Cbr-acbp-4.1, CBG00609, CBG30888, CBG11604, CBG00082
IV:1-4892	IV:1491435-1496326	99.92	Cbr-srv-16.2	CBG05119, Cbr-srv-16.1, CBG27424
IV:578437-584361	IV:584485-590409	99.65	CBG15159, CBG08170	CBG00936
IV:1302973-1306512	IV:11674712-11678275	97.68	Cbr-mesp-1.4, CBG13754	CBG13626, Cbr-maea-1, Cbr-mesp-1.2, Cbr-zgpa-1, CBG05012
IV:1486287-1490434	IV:1589258-1593405	99.97	CBG18719, CBG13798, CBG00797	CBG27430
IV:13351979-13353578	IV:1612007-1613606	99.85	CBG25419	CBG31306, CBG27433
IV:2135188-2138867	IV:4022649-4026444	87.58	CBG21960, CBG21587, Cbr-lbp-4	CBG04213, Cbr-atg-10, Cbr-ceh-49.1, CBG01404
IV:2135429-2136848	IV:2144091-2145584	88.54	CBG21587	Cbr-ceh-49.3
IV:2141648-2144090	IV:4019497-4021943	97.87	CBG21590	CBG30428, CBG01406
IV:2144086-2145798	IV:4016992-4018701	92.51	Cbr-ceh-49.3	Cbr-ceh-49.6
IV:2147784-2150512	IV:4014234-4016992	93.1	CBG30153, CBG21592, CBG20577, CBG16117	CBG15046, CBG01407
IV:2149919-2151085	IV:2135607-2136753	85.39	Cbr-ceh-49.5	CBG21587
IV:2342084-2348827	IV:2334602-2341345	99.99	CBG30494, CBG30495	CBG25182, CBG11716, Cbr-str-33, CBG24588
IV:2906617-2913515	IV:2914002-2920900	99.65	CBG30002, Cbr-srj-32, CBG22430, CBG22431, Cbr-acdh-10, Cbr-acdh-7	Cbr-ptps-1, CBG22233, CBG25832, CBG22432, CBG22435, CBG15943
IV:2883763-2891178	IV:2875332-2882747	98.44	CBG13423, CBG25829, CBG26939, CBG22421, Cbr-sdz-34.4	CBG22223, CBG13424, CBG22357, CBG15951
IV:11132948-11134166	IV:6677825-6679035	99.1	CBG09440	CBG17252
IV:3264186-3267906	IV:3259773-3263493	99.59	CBG13343	CBG31484
IV:3440110-3446474	IV:3462423-3468787	99.76	CBG23889, CBG24733, CBG01928, CBG01929, CBG01931	Cbr-vha-10, CBG00725, CBG30502, CBG10453
IV:3485721-3491767	IV:3536409-3542455	99.48	CBG00719, CBG17492, CBG31482, CBG01942	Cbr-srg-1, CBG22665
IV:3884873-3886206	IV:3881240-3882573	98.25	CBG15020	CBG15019
IV:3881240-3882573	IV:3884873-3886206	98.35	CBG15019	CBG15020
IV:4014317-4017015	IV:2147783-2150512	93.1	CBG15046, CBG01407	CBG30153, CBG21592, CBG20577, CBG16117
IV:4019568-4021944	IV:2141648-2144091	97.43	CBG01406	CBG21590
IV:4022711-4026444	IV:2135148-2138868	87.55	CBG04213, Cbr-atg-10, Cbr-ceh-49.1, CBG01404	CBG21960, CBG21587, Cbr-lbp-4

IV:4824676-4831176	IV:4831529-4838029	99.81	Cbr-knl-1, Cbr-coa-4, CBG31532	CBG31007, CBG31179, Cbr-srj-33, CBG27518, CBG30816
IV:5103394-5106501	IV:11462602-11465725	96.99	Cbr-mcm-4	Cbr-mrpl-48, Cbr-crn-2
IV:5443607-5446884	IV:5438440-5441717	99.9	Cbr-gpx-1, Cbr-gnrr-7	Cbr-cox-7C
IV:5562657-5566988	IV:5691075-5695406	99.56	CBG03773, CBG11185	CBG03730, CBG03731, CBG17501
IV:6012193-6017354	IV:6019155-6024316	99.93	Cbr-vacl-14, CBG29836, Cbr-cul-4, CBG29759, Cbr-cec-6, CBG06319	CBG08223, Cbr-cnep-1, Cbr-mrt-2, CBG17572
IV:6150338-6151836	IV:6154331-6155758	92.98	CBG19895	CBG19894
IV:6154334-6155788	IV:6150335-6151810	92.46	CBG19894	CBG19895
IV:6227912-6230057	IV:6421797-6423912	98.69	CBG08267	CBG18179
IV:6421799-6423909	IV:6227920-6230058	99.81	CBG18179	CBG08267
IV:6464113-6467538	IV:6528469-6531894	99.98	CBG30211	CBG10807, CBG12958, Cbr-skr-17, CBG12956, CBG09465
IV:6848043-6853757	IV:6794784-6800449	98.19	Cbr-pfd-6, CBG18064, Cbr-ipla-6.1	Cbr-xpa-1, CBG03266, Cbr-ipla-6.2, CBG06540, CBG06541
IV:7081315-7082588	IV:7085439-7086739	88.99	CBG30873, CBG05402	CBG05403
IV:7085448-7086739	IV:7081315-7082602	89.52	CBG05403	CBG30873, CBG05402
IV:8209935-8211451	IV:4123483-4124790	99.77	CBG14145	CBG09047
IV:8181097-8182682	IV:6408545-6409915	82.41	CBG05739	CBG19838
IV:7838392-7839970	IV:7694083-7695703	92.03	CBG05839, CBG05838	CBG05886, CBG05885
IV:7836556-7839988	IV:7831202-7834634	99.12	Cbr-cyn-4, CBG05840, CBG05839, CBG05838, CBG08666, Cbr-lin-32	CBG05843, CBG05842, CBG27616
IV:7836556-7838394	IV:7695933-7697771	96.92	CBG05840, CBG08666	Cbr-col-39, CBG05884, CBG29117
IV:7694107-7695703	IV:7833038-7834634	99.12	CBG05886, CBG05885	CBG05842
IV:7830908-7833040	IV:7695933-7698228	96.92	CBG05843	Cbr-col-39, CBG05884, CBG05883, CBG29117
IV:7696959-7698219	IV:8460130-8461347	95.9	CBG05884, CBG05883	CBG06060, CBG06061
IV:7696089-7698218	IV:7830760-7832836	98.32	Cbr-col-39, CBG05884, CBG05883, CBG29117	CBG05844, CBG05843
IV:7696089-7697780	IV:7836556-7838191	98.42	Cbr-col-39, CBG05884, CBG29117	CBG05840
IV:7833037-7834645	IV:7838392-7839999	90.86	CBG05842	CBG05839, CBG05838
IV:7592978-7595000	IV:7590592-7592614	93.85	Cbr-srg-25	CBG12042, CBG16538, Cbr-kbp-1
IV:7397568-7401079	IV:8370163-8373674	99.81	CBG11987, CBG29941, CBG11789	CBG12206, Cbr-ttr-43

IV:8373898-8377633	IV:7393399-7397134	99.88	CBG09753, CBG30417	CBG16488, CBG16489, CBG06702
IV:7224967-7228549	IV:8364722-8368304	99.95	CBG06658	CBG12207, CBG02869, Cbr-best-7.2
IV:8439845-8444977	IV:8452396-8457528	99.88	CBG02845, CBG06055	CBG06057, CBG06058, Cbr-clc-3
IV:8444338-8444977	IV:7694060-7694699	94.01	CBG06055	CBG05886
IV:9188433-9190210	IV:9195134-9196911	99.27	CBG06246	CBG09962, CBG06247
IV:10258416-10264727	IV:10119515-10125826	99.95	Cbr-unc-32, CBG22318, CBG22319, CBG09704, CBG14567	CBG12733, CBG12734, Cbr-ttc-4, Cbr-cdc-14.1, CBG24368, CBG24369, CBG09743, CBG30526, CBG27068
IV:10157466-10158712	IV:10159933-10161177	81.26	CBG06907	CBG27070
IV:10159935-10161177	IV:10157460-10158708	81.18	CBG27070	CBG06907
IV:10285118-10289239	IV:10310865-10314986	99.52	CBG01845, Cbr-scl-15, Cbr-irld-6, Cbr-srh-55.2	Cbr-clec-141, CBG06864, CBG06863, CBG01837
IV:10314590-10315887	IV:10312949-10314246	98.2	CBG01836	CBG06863, CBG01837
IV:10520075-10521242	IV:10521845-10523013	95.93	CBG01768	CBG01767
IV:10521846-10523014	IV:10520073-10521241	96.01	CBG01767	CBG01768
IV:10306609-10308049	IV:10729123-10730563	100	CBG25644, CBG01838	CBG01707
IV:11051208-11052589	IV:11054362-11055742	92.41	CBG29880, CBG01630	CBG01629
IV:11054362-11055823	IV:11051208-11052589	92.26	CBG01629	CBG29880, CBG01630
IV:11138532-11139817	IV:11598259-11599460	96.94	CBG03615	CBG20840
IV:11339388-11346596	IV:11347597-11354805	99.99	CBG08476, CBG08475, Cbr-kin-5.1, Cbr-nlp-10, CBG31347, CBG09397, Cbr-inx-3.1, CBG14795, CBG14796	CBG20777, Cbr-dmsr-7, CBG09394, CBG09393, CBG14797, CBG14798
IV:11462602-11465725	IV:5103393-5106502	96.83	Cbr-mrpl-48, Cbr-crn-2	Cbr-mcm-4
IV:11598257-11599461	IV:11138552-11139755	96.94	CBG20840	CBG03615
IV:13738028-13739370	IV:13652242-13653583	97.18	CBG18475	CBG18462
IV:14199703-14203180	IV:6748366-6751865	98.17	CBG10348, Cbr-irld-14, Cbr-str-124	CBG29843

IV:14204214-14205410	IV:14669012-14670261	94.4	CBG03512	CBG16471, Cbr-rpoa-12
IV:14373173-14378476	IV:14378577-14383880	99.87	CBG10294, Cbr-pssy-2, CBG04548	CBG10292, Cbr-oig-3, Cbr-unc-61
IV:15073694-15080663	IV:15065737-15072710	99.93	Cbr-cht-2	Cbr-mrps-18B
IV:15864423-15868944	IV:15869987-15874508	99.31	CBG07153, CBG31454, Cbr-srd-21	CBG13950, CBG04993, CBG15449
IV:16334530-16337091	IV:16447608-16450174	99.69	CBG07048	CBG30392, CBG23981
IV:16632589-16638046	IV:16594964-16600421	98.27	CBG30987, CBG27998	CBG06960, CBG10577, Cbr-vamp-7, CBG24406
IV:17221319-17225744	IV:17212941-17217366	99.73	CBG26539, CBG30908	CBG13515
V:599181-600665	V:623616-625191	80.69	CBG08173	CBG08179
V:999067-1003150	V:1003284-1007367	99.9	CBG13705, CBG16370	Cbr-sdhb-1, CBG30300, CBG13706
V:1250682-1251830	V:1239196-1240370	98.89	CBG01506, CBG01507	CBG26907, CBG01498
V:1248721-1249878	V:1241031-1242217	97.48	CBG01504, Cbr-his-63	CBG01499, CBG01500
V:1241052-1242163	V:1248749-1249880	98.42	CBG01499, CBG01500	CBG01504, Cbr-his-63
V:1239217-1240366	V:1250659-1251832	98.89	CBG26907, CBG01498	CBG01506, CBG01507
V:1615509-1617177	V:6736547-6738217	96.99	CBG27434	CBG06519
V:1793491-1798178	V:1799932-1804619	99.34	Cbr-tufm-1, CBG30329, CBG26240, Cbr-nmat-1	Cbr-rps-18
V:11475297-11476910	V:7473277-7474891	98.39	CBG26451	CBG26804
V:2002577-2006499	V:4429198-4433114	98.24	CBG21557, CBG31291, CBG20545	Cbr-cytb-5.2, Cbr-dhod-1, CBG01282, CBG31338, CBG27508
V:2591538-2598335	V:2647224-2654021	99.9	CBG13477, CBG21823	Cbr-clec-137, CBG13463, CBG21836, CBG21837, CBG15990
V:2726539-2730247	V:2479336-2483044	99.91	CBG26113, Cbr-srbc-59	CBG31243
V:3152977-3157304	V:3148108-3152435	99.6	CBG17014	CBG27473
V:3053644-3057499	V:3049778-3053633	99.88	CBG22265, CBG13383, CBG13382, CBG31198, CBG16979, CBG31269	CBG16978
V:3308773-3310341	V:7286915-7288488	98.6	CBG13335	CBG30215, CBG17135
V:3484959-3486836	V:3490509-3492386	99.73	CBG10446	CBG10445
V:3717153-3720587	V:3551416-3554850	99.99	CBG01988, CBG30710, CBG01989, CBG27487	Cbr-wdr-12, CBG10431, CBG10430
V:3576941-3578198	V:3579150-3580410	95.75	CBG10424	CBG10423
V:3579150-3580409	V:3576941-3578199	95.83	CBG10423	CBG10424
V:4256370-4257436	V:4261518-4262553	84.98	CBG00569	CBG01340

V:4429253-4432344	V:2003348-2006498	95.4	Cbr-cytb-5.2, CBG01282, CBG31338, CBG27508	CBG21557, CBG31291, CBG20545
V:4534984-4539853	V:4543559-4548439	97.59	CBG30453, CBG01247, Cbr-nhr-190.1	CBG21747, CBG01244
V:4710542-4711672	V:4732294-4733424	97.62	CBG01197	CBG01188
V:4727914-4729992	V:4714693-4716780	96.69	CBG01191	CBG01195, CBG01194
V:1591124-1595255	V:7018817-7022939	98.87	CBG15784	Cbr-mab-21, CBG26374
V:4963465-4967087	V:4748484-4752106	99.2	CBG03918, CBG29829, Cbr-hsp-12.3	CBG25242, CBG21719
V:4986537-4988441	V:4991276-4993180	98.53	CBG01114	CBG03913, CBG01112
V:5443652-5448955	V:5493285-5498587	99.27	Cbr-gpx-1, CBG20035, Cbr-gnrr-7	Cbr-nubp-1, CBG30963, CBG18258, Cbr-elo-7, CBG27003
V:5553517-5555138	V:5548397-5550061	96	Cbr-nhr-247.2	CBG03776, Cbr-nhr-247.1
V:5548677-5550060	V:5553517-5554908	98.64	CBG03776, Cbr-nhr-247.1	Cbr-nhr-247.2
V:5712617-5717525	V:5877267-5882132	98.66	CBG25223, Cbr-marc-3, CBG29835, CBG17888, Cbr-lpr-5	CBG26570, CBG13096, CBG13192, Cbr-pepm-1.1, CBG30594, CBG17941
V:5873554-5874720	V:5882233-5883412	99.07	CBG17939	CBG06277
V:6097873-6101801	V:7009895-7013828	99.97	CBG06338	Cbr-erh-2, CBG05425, CBG06602
V:6102339-6106551	V:7013985-7018197	99.88	CBG13251, CBG19910, CBG06339, CBG06340	Cbr-asb-1, Cbr-srbc-36, CBG06605
V:6632625-6634060	V:6630383-6631817	99.79	CBG06483	CBG06482
V:6692003-6699160	V:6699653-6706810	99.65	CBG20490, CBG03287, CBG30480, CBG05511, CBG30902, CBG06506	CBG20487, Cbr-best-23, CBG06508, CBG30903, CBG06509, CBG31518
V:6792053-6797063	V:17754576-17759614	96.77	CBG05481, Cbr-srw-103, CBG06539, CBG17232	CBG17824
V:6798082-6807842	V:17742713-17752578	96.99	Cbr-crml-1, Cbr-dnj-23, Cbr-dyrb-1, CBG03263, Cbr-txt-9.2, Cbr-mnp-1, CBG26378, Cbr-ops-1, CBG06541, CBG06542, Cbr-gur-3	CBG17823, CBG30845, CBG30844, Cbr-srw-101.1
V:6805543-6808207	V:17742351-17745029	96.69	Cbr-ops-1	CBG17823
V:6828374-6830854	V:17738590-17741140	95.12	CBG06553	CBG17821
V:7000583-7008876	V:7024096-7032389	99.86	Cbr-col-61, CBG18035, CBG30448, CBG31384, Cbr-ddi-1, CBG05426, CBG06598, CBG06599, CBG06600, CBG06601	CBG21944, CBG11902, CBG11903, Cbr-fan-1, Cbr-paqr-1, CBG06608, CBG06609, CBG06610, Cbr-nhr-178
V:7069686-7079545	V:7503125-7512985	99.69	CBG03194, Cbr-odr-8, CBG05409, Cbr-dnj-11,	Cbr-glh-4, CBG25567, CBG03087, CBG03086, CBG16518, Cbr-tofu-7

			Cbr-lec-11, CBG05406, CBG17182	
V:7286918-7288488	V:3308773-3310341	98.54	CBG30215, CBG17135	CBG13335
V:7473275-7474892	V:5830803-5832411	99.38	CBG26804	CBG25854
V:7496549-7497800	V:12647783-12649040	99.44	Cbr-tmem-17	CBG10990
V:7796605-7800344	V:7808914-7812661	99.92	CBG12082, Cbr-srab-3.2, CBG14047	CBG12086, CBG25575, CBG25932, Cbr-srab-3.1
V:7802613-7808915	V:7812923-7819227	99.92	Cbr-aars-2, CBG31425, Cbr-crn-4	CBG12088, Cbr-mrps-6, CBG03007, CBG05848, CBG08671
V:7839233-7840526	V:3635518-3636828	99.01	CBG05838	Cbr-col-176
V:8035783-8039182	V:8323384-8326783	99.97	CBG25104, CBG25105, Cbr-mutd-1, CBG08604, CBG31468	Cbr-wdr-5.1, CBG24764, CBG24765
V:8688628-8690761	V:8696394-8698533	98.98	CBG23445, CBG23444	Cbr-col-86, CBG23437, CBG23436
V:8690871-8692346	V:8694903-8696394	97.39	CBG23443, CBG23442	CBG23438
V:8694917-8696395	V:8690860-8692345	98.26	CBG23438	CBG23443, CBG23442
V:8696407-8698533	V:8688617-8690762	98.79	Cbr-col-86, CBG23437, CBG23436	CBG23445, CBG23444
V:9396376-9399738	V:9402167-9405544	99.97	Cbr-unc-120	CBG10027
V:9399708-9402130	V:9409565-9411987	99.93	Cbr-mpst-1.2	CBG29920, Cbr-set-4, CBG03338, CBG03339, Cbr-mpst-1.1
V:9549597-9550606	V:9646412-9647413	99.6	Cbr-col-157.2	Cbr-col-157.1
V:9760395-9763760	V:8340151-8343516	94.88	CBG02546	CBG12210, CBG02877
V:8340162-8342434	V:8343618-8345890	95.12	CBG02877	CBG02875
V:8353245-8358744	V:9763771-9769270	99.96	CBG05701, CBG27022, CBG31220	CBG02545, CBG02544, CBG30848, CBG10123, CBG03424, CBG30653
V:10121964-10125612	V:10159335-10162983	99.87	CBG09743, CBG30526, CBG27068	CBG24380, Cbr-dod-24, CBG27070, Cbr-sec-61.G
V:10265605-10266901	V:14102969-14104290	93.06	CBG09703	CBG11554
V:11052533-11060615	V:11060716-11068798	99.98	CBG20245, CBG20667, Cbr-gst-12, CBG01629, CBG01628, CBG01627, CBG29115	Cbr-gst-15, Cbr-gst-24, Cbr-gst-20, Cbr-csc-1, Cbr-fbxc-23, CBG03602, CBG01626, CBG01624, CBG26446
V:11134458-11140118	V:11128430-11134089	99.95	CBG03615, CBG14754	CBG08517, Cbr-max-1.2, CBG09440
V:12476774-12480759	V:12482518-12486503	99.52	CBG20423, CBG19636, CBG12261, CBG19023	CBG30245, CBG26494, Cbr-dmsr-11, Cbr-cah-5
V:12600724-12604044	V:13133560-13136877	99.91	CBG26489	CBG04336

V:12604039-12614128	V:13136872-13146956	99.76	CBG18795, Cbr-nhr-88, CBG12283, CBG26488, CBG30366, CBG30367, CBG19050, CBG19052, CBG19053, Cbr-alh-10	CBG15859, CBG15858, CBG15857, CBG04338, CBG25522, Cbr-gop-2, Cbr-col-103, CBG26472, CBG19209, Cbr-srx-16.2, Cbr-srx-16.1, Cbr-pcyt-1, CBG16734
V:12792386-12798751	V:12803727-12810092	99.78	Cbr-set-11, CBG19561, CBG27169, Cbr-hpo-9, Cbr-hyls-1.1, Cbr-spp-2, Cbr-spp-5, CBG27790, CBG27791	CBG26597, CBG19559, CBG19558, CBG19557, CBG21241, CBG19115, CBG19116, Cbr-hyls-1.2
V:13435507-13438945	V:14044466-14047904	99.13	CBG19274	CBG19434
V:14075721-14077736	V:14533141-14535156	96.95	CBG30297	CBG24814
V:14284794-14286909	V:14364904-14366860	86.38	CBG11614	CBG10301, CBG04543
V:14364989-14366831	V:14284809-14286878	85.28	CBG10301, CBG04543	CBG11614
V:14581636-14583534	V:14635555-14637499	93.79	CBG04591	CBG04607, CBG04608
V:14635538-14637610	V:14581641-14583613	94.8	CBG04607, CBG04608	CBG04591, CBG04592
V:15337441-15338195	V:15335630-15336384	98.76	CBG04806	CBG04804
V:14928785-14929720	V:14904066-14905001	95.86	CBG04710	CBG04696
V:14905700-14908099	V:14902697-14905095	97.33	CBG04698, CBG00220	CBG04695, CBG04696
V:14902678-14905095	V:14905698-14908098	96.42	CBG04695, CBG04696	CBG04698, CBG00220
V:15595271-15598316	V:15521456-15524559	98.11	CBG04880	CBG04857, CBG31263
V:15599123-15600191	V:15644224-15645317	95.26	CBG04882, CBG30796	CBG04902
V:15644224-15645318	V:15519513-15520607	95.75	CBG04902	CBG04855
V:15511106-15517027	V:15602224-15608145	97.43	CBG23572, CBG04852, CBG04853, CBG15530	CBG30089, CBG04884, CBG04885
V:15512562-15515972	V:15640702-15644116	97	CBG04852, CBG04853, CBG15530	CBG04904
V:15695416-15698154	V:15704415-15707157	96.03	CBG30090, CBG04923, CBG27260	CBG04926, CBG31266, CBG27968
V:15782678-15784028	V:15784308-15785658	97.2	CBG04955	CBG04956
V:15784333-15785704	V:15782674-15784028	97.3	CBG04956	CBG04955
V:16560669-16561924	V:16567634-16568855	81.47	CBG16823, CBG24668	CBG24669
V:16577531-16582375	V:16571697-16576541	99.13	Cbr-nstp-4, CBG22558, CBG24401	CBG24357, CBG22733
V:16662858-16669610	V:16746600-16753359	99.87	CBG30395, CBG22717	CBG24530, CBG22701
V:16754883-16756647	V:16761860-16763588	94.75	CBG22915	CBG22913
V:16761860-16763584	V:16754923-16756649	94.81	CBG22913	CBG22915
V:16852460-16853928	V:16885006-16886475	97.89	CBG22885	CBG22876
V:16873731-16877972	V:16920531-16924772	98.54	CBG22880, CBG22879	CBG22500, CBG22501, CBG22867, CBG05232
V:16924286-16927858	V:16877021-16880659	99.31	CBG22865, CBG05233	CBG22878, CBG22877

V:16925393-16929958	V:16930960-16935525	99.16	CBG22865, CBG22864, CBG22863	CBG26532, CBG22862, CBG22861, CBG22860, CBG05235
V:16877021-16879486	V:16930960-16933425	99.16	CBG22878	CBG22862
V:16971167-16972718	V:16972967-16974518	96	CBG22843	CBG22842
V:16998066-17001663	V:17016446-17020043	97.96	CBG30398, CBG31228, CBG31227	CBG22516, CBG22832
V:17194835-17197015	V:17217104-17219283	98.44	CBG18656, CBG28021	CBG18650
V:17197171-17200946	V:17219379-17223154	99.17	CBG13511	CBG26539, CBG18649
V:17252822-17257888	V:17257889-17262955	99.92	Cbr-ham-1, Cbr-str-96.1, CBG28028, CBG05310	CBG18638, Cbr-str-96.2, CBG28029
V:17409522-17411096	V:11475297-11476915	98.64	Cbr-ced-2	CBG26451
V:17738592-17741135	V:6828360-6830856	95.28	CBG17821	CBG06553
V:17742353-17746622	V:6803938-6808211	97.56	CBG17823, CBG30845	Cbr-clh-5, Cbr-ops-1
V:6798057-6803886	V:17746736-17752565	99.21	Cbr-dnj-23, Cbr-dyrb-1, Cbr-txt-9.2, CBG26378, CBG06541, Cbr-gur-3	CBG30844, Cbr-srw-101.1
V:17754557-17759631	V:6792052-6797090	96.75	CBG17824	CBG05481, Cbr-srw-103, CBG06539, CBG17232
V:18073829-18080269	V:18081971-18088411	99.88	CBG23817, CBG23818, CBG07673	CBG27335, CBG23820, CBG07671
V:19227783-19232321	V:19232520-19237058	99.92	Cbr-snx-6	CBG31387, CBG28079, Cbr-cdk-4
V:19348264-19355264	V:19356265-19363265	99.91	Cbr-str-93	CBG05675, Cbr-nlp-53, CBG07404
X:655838-664603	X:591827-600592	99.64	CBG11342, CBG26883, Cbr-ife-2.2	Cbr-dpl-1, CBG13652, CBG11356, CBG31495, Cbr-ife-2.1, CBG30827, CBG08173
X:470451-474130	X:1436403-1440055	98.03	CBG15181, CBG11389	Cbr-cutl-20, CBG26913, CBG00777, CBG16276
X:1436403-1440049	X:6856935-6860571	98.44	Cbr-cutl-20, CBG26913, CBG00777, CBG16276	CBG25467, CBG21906, Cbr-nex-3, Cbr-cnc-11, Cbr-cnc-4
X:160068-161678	X:5494157-5495765	95.77	Cbr-arrd-4	CBG30963
X:1111674-1119550	X:927943-935819	99	CBG29802, CBG29803, CBG29804, Cbr-cus-2, Cbr-nlp-63, CBG01464, CBG31299, Cbr-acd-4, CBG16347	Cbr-strm-1, CBG13699, Cbr-gpa-2
X:1436127-1440008	X:6856933-6860845	98.12	Cbr-cutl-20, CBG00775, CBG26913, CBG16276	CBG25467, CBG21906, Cbr-nex-3, Cbr-cnc-11, Cbr-cnc-4, Cbr-cnc-3
X:1436403-1440013	X:470479-474129	97.96	Cbr-cutl-20, CBG26913, CBG16276	CBG15181, CBG11389

X:1766561-1771904	X:1771915-1777258	99.03	CBG22016	CBG18442
X:2370428-2376403	X:2376575-2382550	99.92	Cbr-elc-1, CBG11706, CBG23597, CBG23599, CBG16068	CBG23602, CBG23603, CBG23605, CBG16067
X:2437112-2444649	X:2445650-2453187	99.78	Cbr-bpnt-1, Cbr-pstk-1, CBG18739, CBG26260, CBG31211	CBG31214, CBG31215, CBG31216, CBG16054
X:3047844-3049586	X:3045681-3047423	91.69	CBG16977	CBG22262
X:3443033-3445309	X:3445816-3448095	99.87	CBG01929, CBG01931	CBG14897, CBG01932, CBG01933
X:3445814-3448089	X:3443036-3445316	99.78	CBG14897, CBG01932, CBG01933	CBG01929, CBG01931
X:3714048-3716038	X:3721080-3723070	99.55	CBG30709	CBG19690
X:3721082-3723080	X:3714049-3716038	99.95	CBG19690	CBG30709
X:4595454-4598017	X:4590125-4592679	96.88	Cbr-elof-1, CBG02162	CBG11101, CBG02161
X:4740312-4741373	X:4738999-4740060	97.87	CBG26290	CBG01185
X:4899445-4905960	X:4906961-4913476	98.92	CBG30895	CBG03932, CBG01134
X:5122661-5126341	X:5127342-5131022	99.49	Cbr-tbca-1, Cbr-hpr-9	CBG27527
X:5494141-5495683	X:160065-161678	95.4	CBG30963	Cbr-arrd-4
X:5697376-5702158	X:5664184-5668965	98.62	Cbr-snpn-1, CBG30837, CBG17503	CBG26416, CBG17881, CBG17448, CBG31519
X:6289151-6292122	X:6293261-6296232	99.63	CBG04526	CBG04525
X:6618586-6620661	X:6698713-6700787	99.38	CBG26631, CBG18125, CBG31402	CBG29924, CBG06508
X:6618059-6620664	X:6698713-6701318	99.61	CBG26631, CBG18125, CBG31402, CBG17262	CBG29924, CBG20487, CBG06508
X:6856874-6860876	X:1436127-1440050	98.09	CBG25467, CBG21906, Cbr-nex-3, Cbr-cnc-5.1, Cbr-cnc-11, Cbr-cnc-4, Cbr-cnc-3	Cbr-cutl-20, CBG00775, CBG26913, CBG00777, CBG16276
X:7259860-7262600	X:7265937-7268677	97.58	Cbr-clec-156.2, CBG17143, CBG17142	CBG11950, CBG17140, CBG17139
X:7256685-7261799	X:7262714-7267881	98.88	CBG31491, Cbr-clec-156.1, Cbr-clec-156.2, CBG17143, CBG17142	CBG11949, CBG03138, CBG17140, CBG17139
X:6681205-6684273	X:7288073-7291259	99.28	CBG31260	CBG17956, CBG17135
X:7373724-7376602	X:7284195-7287072	99.13	Cbr-abf-1	Cbr-spo-11
X:7515512-7517699	X:7440018-7442180	96.44	CBG26807	CBG03104, CBG11803
X:7514141-7517487	X:7497578-7500925	99.88	CBG03083, CBG26807	Cbr-lin-37, Cbr-flp-13, CBG11821, CBG11822
X:7440202-7442180	X:7498947-7500925	99.88	CBG03104, CBG11803	CBG11822

X:7449732-7454768	X:7443695-7448731	99.72	Cbr-pmlr-1, Cbr-clec-143.2, CBG05958, Cbr-cutl-1, CBG11808	CBG29846, CBG11998, CBG29847, CBG03103, CBG06720, CBG11805
X:7498950-7500925	X:7515510-7517485	97.4	CBG11822	CBG26807
X:7503651-7506695	X:7388016-7391057	99.87	CBG03087	CBG05975
X:7619401-7620961	X:7625688-7627251	93.87	CBG27610	CBG03052
X:7625690-7627251	X:7619401-7620960	94.12	CBG03052	CBG27610
X:7748515-7752456	X:9550084-9554025	99.75	Cbr-npp-19, CBG14035, CBG14036	CBG30838, Cbr-rbmx-2, Cbr-cpb-2, CBG10073, CBG27058, CBG23217, CBG14426, CBG14427
X:9646665-9655132	X:7765137-7773604	99.96	Cbr-cuti-1, CBG12616, Cbr-rbbp-5, CBG10096, CBG10097, CBG03396, CBG23184, CBG14454	CBG31426, Cbr-srh-130, CBG27004, Cbr-srh-129, CBG14040
X:8271266-8276985	X:8282749-8288468	99.95	CBG12234, CBG02903, CBG31023	CBG12230, CBG25291, CBG02900, CBG26367, Cbr-egg-1
X:8365343-8367845	X:8356871-8359369	99.2	CBG12207	CBG31220
X:8483578-8487333	X:8685095-8688858	96.13	CBG29852, CBG29853, Cbr-gspd-1	Cbr-srh-16
X:8685090-8688854	X:8483561-8487320	96.49	Cbr-srh-16	CBG29852, CBG29853, Cbr-gspd-1
X:9369751-9372074	X:9656133-9658448	100	CBG12535	Cbr-nlp-48, CBG23182
X:9487031-9490039	X:9491391-9494392	99.87	CBG25907, Cbr-vrp-1, CBG27678	Cbr-blos-4, CBG10053, CBG14412
X:9491382-9494391	X:9487041-9490040	99.87	Cbr-blos-4, CBG10053, CBG14412	CBG25907, Cbr-vrp-1, CBG27678
X:9856224-9863089	X:9867880-9874745	99.96	CBG02525, CBG14491, Cbr-amt-4.1	CBG02522, Cbr-dhs-12, Cbr-gst-2, Cbr-gst-4, CBG03453, CBG14493
X:10156562-10160143	X:10588151-10591732	99.89	CBG25368, CBG06907, CBG24379	CBG30140, Cbr-srd-10
X:10590363-10594065	X:10603200-10606902	99.6	CBG25653	Cbr-irld-34, CBG01748
X:10585966-10587150	X:10615451-10616635	99.94	CBG02324, CBG14645	CBG14647
X:10851780-10859904	X:11347480-11355604	99.95	CBG12895, Cbr-nep-12, Cbr-rpl-7A, Cbr-tald-1, Cbr-srsx-30, Cbr-srsx-29, CBG14696, CBG14697	CBG20777, Cbr-rei-1, Cbr-dmsr-7, CBG09394, CBG09393, CBG30687, CBG14797, CBG14798
X:11136277-11137206	X:11140063-11140992	99.68	CBG14754	CBG14755
X:11340033-11346458	X:11416919-11423344	99.88	CBG08476, CBG08475, Cbr-kin-5.1, Cbr-nlp-10, CBG31347, Cbr-inx-3.1, CBG14795	CBG25399, CBG25676, CBG30239, CBG20201, CBG09375, Cbr-cox-16, Cbr-inx-3.2, CBG14812
X:12756679-12761394	X:12761613-12766328	99.84	CBG30851, CBG19101	CBG08316

X:13349711-13353353	X:13353440-13357082	99.86	CBG29972, CBG17758	CBG17755
X:14045022-14047859	X:13547940-13550777	97.46	CBG19434	CBG26193, CBG19306, Cbr-nstp-10
X:15027347-15032984	X:15033985-15039622	99.95	CBG21494, CBG00244	Cbr-str-52, CBG00245
X:15839420-15840722	X:15840923-15842249	91.29	CBG30555, CBG15457	CBG15456
X:15840932-15842249	X:15839411-15840722	91.14	CBG15456	CBG30555, CBG15457
X:16808152-16809399	X:16816156-16817403	99.05	CBG22684	CBG27301, CBG22683
X:17268644-17272126	X:17274419-17277868	99.65	Cbr-manf-1	Cbr-dml-1, CBG18632, CBG05315
X:17274061-17277870	X:17268677-17272484	99.71	Cbr-dml-1, CBG18632, CBG05315	Cbr-manf-1
X:18945464-18947851	X:19079583-19081966	99.71	CBG07494	CBG05615, CBG07468
X:19561527-19563294	X:13028350-13030054	95.98	CBG07374	CBG30545, CBG16706
X:20776916-20778335	X:20568522-20569942	90.13	CBG10263	CBG10217
X:20681624-20683631	X:21078696-21080710	98.66	CBG10247	CBG08890
X:20568523-20570003	X:20776909-20778314	90.76	CBG10217	CBG10263
X:20565780-20569732	X:20557825-20561777	98.13	CBG10216, CBG10217	CBG10213
X:20557825-20561793	X:20565780-20569729	97.7	CBG10213	CBG10216, CBG10217
X:21115273-21117755	X:20882283-20884765	99.76	CBG08881	CBG08942
X:21078697-21080797	X:20681620-20683632	98.61	CBG08890	CBG10247
X:21169837-21171898	X:21537391-21539452	97.67	CBG10674	CBG10579
X:21149080-21156938	X:21189823-21197681	99.95	CBG10681, CBG10680, CBG31485	CBG10666, CBG10665, CBG10664
X:21438766-21440732	X:21444674-21446639	99.75	CBG10602, CBG10601	CBG10598, CBG10597
X:21443123-21444679	X:21440831-21442387	100	CBG10599	CBG10600
X:21445506-21446609	X:21438767-21439898	99.91	CBG10597	CBG10602
